# Supplementary material for: Newborn Screening Protocols and Positive Predictive Value for Congenital Adrenal Hyperplasia Vary across the United States
Source: Int J Neonatal Screen. 2020 May 8;6(2):37. doi: 10.3390/ijns6020037 (PMC7422998; doi:10.3390/ijns6020037)
Supplement: Supplementary file 1 [file IJNS-06-00037-s001.pdf]

Newborn screening procedures CAH questionnaire

Newborn screening, initial assessment and follow up for abnormal 17-hydroxyprogesterone in the US.

1. **Baseline epidemiologic data**:
    a. # newborns screened/state annually
    b. # positive screens annually
    c. # positive screens in premature infants (define gestational age considered premie) annually
    d. # confirmed cases annually
    e. # that were not closed (lost to follow up)/year
    f. Is there a length of time for lost to follow up?
    g. If so, what is that time?

2. **State lab screening methods:**
    a. State lab based or send out (i.e. Perkins Elmer for example)?
    b. What specific assay is used?
    c. What is your reference range or cut-point for 17OHP for…?
        1. Full-term newborns?
        2. By gestational age?
        3. By birthweight?
        4. Other?
    d. Does your state utilize other analytes or ratios in making the diagnosis of CAH-21OH def?
        1. If so, what analytes or ratios are used?
        2. Cut-points for those measurements?
    e. Does your state mandate a second newborn screen?
        1. If so, what method is used?

3. **Coordinators:**
    a. Do you have a newborn screening coordinator?
        1. If so, who is the coordinator (administrative person, nurse, endocrinologist, other)?
        2. In what department is the coordinator based (endo, genetics, other)?
        3. Are they responsible only for endocrine results?
        4. How much dedicated time do they have to perform their role as coordinator?

4. **Initial screen:**
    a. To whom are results of the initial CAH NB screen communicated (coordinator, PCP, endocrinologist, other)?
    b. Is this communicated differently during nights, weekends or holidays?
    c. By whom are results of the initial screen communicated?
    d. How are results of the initial normal screen communicated (fax, email, call)?
    e. How are results of the initial critical abnormal screen communicated (fax, email, call)?
        1. How are results of the initial quantity insufficient screen communicated (fax, email, call)?

5. **Follow up of abnormal initial screen- newborn has been discharged from the hospital**
    a. Who contacts the family with abnormal screen results (PCP, coordinator, endocrinologist?
    b. For abnormal screen, the protocol is to:
        1. repeat screen
        2. to repeat screen and have electrolytes drawn
        3. to repeat screen and have electrolytes and 17 OHP drawn
        4. there is no protocol; at discretion of provider
    c. When the family is asked to repeat newborn screen. They are instructed to go to (check all that apply):
        1. local health department
        2. a draw site
        3. PCP office
        4. Ped endo office
        5. local hospital
        6. any of the above
    d. A pediatric endocrinologist is called for (check all that apply):
        1. Abnormal screen
        2. Critical screen
        3. Abnormal electrolytes
        4. Results of an abnormal repeat serum 17-hydroxyprogesterone

6. **Follow up of abnormal screen-newborn is in hospital:**
    a. Who contacts the hospital with abnormal screen results? And how? phone call/fax?
    b. Who is contacted in the hospital (administrative person, bedside nurse, hospital charge nurse, nurse practitioner, fellow or attending neonatologist, pediatric endocrinologist, other)?
    c. Is the newborn screening coordinator also contacted, if one exists?
    d. For abnormal screen in the hospital, the protocol is to:
        1. repeat screen
        2. to repeat screen and have electrolytes drawn
        3. to repeat screen and have electrolytes and 17 OHP drawn
        4. there is no protocol; at discretion of provider
    e. A pediatric endocrinologist is called for (check all that apply):
        1. Abnormal screen
        2. Critical screen
        3. Abnormal electrolytes
        4. Results of an abnormal repeat serum 17-hydroxyprogesterone

7. **Follow up of patient discharged from the hospital or moved before newborn screening follow up is complete.**
    a. Who is responsible for ensuring follow up of NBS in the outpatient setting?
